# Current challenges in understanding immune cell functions during septic syndromes

**DOI:** 10.1186/s12865-015-0073-4

**Published:** 2015-03-26

**Authors:** Zechariah Franks, McKenzie Carlisle, Matthew T Rondina

**Affiliations:** Program in Molecular Medicine, Salt Lake City, 84112, Utah USA; Division of General Internal Medicine, University of Utah School of Medicine, Salt Lake City, 84112, Utah USA

**Keywords:** Sepsis, Neutrophils, Dendritic cells, Infection, Inflammation, Immunity

## Abstract

**Background:**

Sepsis is a dynamic infectious disease syndrome characterized by dysregulated inflammatory responses.

**Results:**

Despite decades of research, improvements in the treatment of sepsis have been modest. These limited advances are likely due, in part, to multiple factors, including substantial heterogeneity in septic syndromes, significant knowledge gaps in our understanding of how immune cells function in sepsis, and limitations in animal models that accurately recapitulate the human septic milieu. The goal of this brief review is to describe current challenges in understanding immune cell functions during sepsis. We also provide a framework to guide scientists and clinicians in research and patient care as they strive to better understand dysregulated cell responses during sepsis.

**Conclusions:**

Additional, well-designed translational studies in sepsis are critical for enhancing our understanding of the role of immune cells in sepsis.

## Review

Despite decades of molecular, clinical, and translational research, sepsis remains a significant public health burden in the United States and worldwide. More than 750,000 patients with sepsis, severe sepsis, or septic shock are admitted into United States hospitals annually and this number continues to rise each decade [1]. Unfortunately, adverse outcomes following septic syndromes remain only marginally improved [2]. Many of the improvements in sepsis management are attributable to a better understanding of appropriate processes of care, such as “bundling”, ventilator management, and goal-directed therapy [3]. Advances in sepsis treatment as a result of improved therapeutic agents have been more modest. In addition, mortality and other outcome estimates are complicated by heterogeneous definitions of illness severity and organ dysfunction, increased surveillance for sepsis, and changes in electronic coding to capture the diagnosis of sepsis [4].

Sepsis is also commonly associated with a number of longer-term complications, including cognitive dysfunction, debilitation, and significant reductions in health-related quality of life in patients who survive sepsis [5-7]. These adverse longer-term outcomes are especially common in the elderly. As the risk and incidence of sepsis increases with age, coupled with forecasts of a sustained rise in the age of the population, septic syndromes will continue to be a common and substantial public health issue [8,9]. As such, ongoing research efforts examining the fundamental cellular and biological mechanisms underlying septic physiology are needed.

These limited successes in the management of septic syndromes are not due to lack of effort. Through ongoing, innovative, and rigorous scientific inquiry, the field has seen the development of advances in diagnostic and prognostic biomarkers and scoring systems, promising pre-clinical animal studies, and a substantial number of clinical trials testing therapeutic agents targeting thrombo-inflammatory mediators and pathways. Despite these efforts, only a few therapeutic agents made it to phase III clinical trials and none have seen sustained clinical use. For example, two of the most promising therapeutics recently met unfortunate endings: activated protein C (APC) was pulled from the market and an anti-toll-like-receptor 4 compound failed in a phase III clinical trial [10]. While investigators continue to identify and study new therapies that hold promise, there is a growing body of evidence suggesting that single therapeutic agents may not be an effective solution for a dynamic, complicated disease like sepsis [11]. The end result of these and other setbacks illustrates that we are still fundamentally limited in our understanding of immune system dysregulation, cell-pathogen interactions, and safe and effective therapies to modulate injurious responses during septic syndromes. The goal of this brief review is to describe current challenges in understanding immune cell functions during sepsis. We also provide a framework to guide scientists and clinicians in research and patient care as they strive to better understand dysregulated cell responses during sepsis. For additional, well-written, and comprehensive reviews on individual aspects of sepsis, the reader is referred to other recent publications [12,13].

### Sepsis is a dynamic, heterogeneous disease process in humans

Sepsis remains a highly complex, heterogeneous, and dynamic disease process in humans. Differences in pathogen virulence, clinical presentations, and individual patient responses to bacterial and viral invaders make sepsis in humans a challenging disease to study. Moreover, certain patient groups are at much higher risk for sepsis. For example, the incidence of sepsis is disproportionately higher in the elderly, and age is an independent predictor of sepsis-related mortality. While comprising only 12% of the US population, older individuals aged ≥65 years represent approximately 65% of all sepsis cases [14]. Older sepsis non-survivors die earlier during hospitalization compared with younger non-survivors. In addition, and complicating efforts to study age-related immune responses in sepsis, older septic patients are often immunologically impaired prior to the development of sepsis due to comorbid illnesses and are thus more susceptible to infection and subsequent complications [15-17]. For those older patients who survive, they require more skilled nursing or rehabilitative care after hospitalization than younger sepsis survivors. This increased risk of sepsis, death, and associated adverse outcomes in older patients, while incompletely understood, may partially be due to immunosenescence, or age-related impairment of inflammatory responses and immune system functions [17-19].

Premorbid factors modify both the disease process and therapeutic approaches used during the course of sepsis. Premorbid factors also contribute to heterogeneity in disease severity, cellular immune functioning, and the safety and effectiveness of therapeutic agents studied for sepsis. For example, an investigation using a global registry of over 12,000 patients with severe septic shock found that diabetes (23%), chronic lung disease (17%), active cancer (16%), congestive heart failure (14%), renal insufficiency (11%), and liver disease (7%) were common comorbidities [20]. Immunologic comorbidities such as immune suppression, cancer, HIV/AIDS, and hepatic failure are also risk factors for sepsis-related mortality [6,21]. Intriguingly, obesity has been associated with improved mortality among severe sepsis patients [22].

Genetic variations may also influence susceptibility to sepsis. In a landmark study of adoptees, premature death in adopted adults had a large heritable component, especially infectious-related death [23]. These, and other investigations, suggest that genetic factors may play an important role in determining the risk of sepsis and sepsis-related adverse outcomes, such as mortality. Nevertheless, many questions remain regarding the contribution of genetics to the risk of sepsis, and it is likely that any genetic factor is polygenic, such that multiple genetic variants are involved [24,25].

### Sepsis is a dynamic disorder of dysregulated inflammatory and immune responses

Many factors limit advances in our understanding of immune cell functions in sepsis. One factor is the evolving appreciation that sepsis is a much more dynamic process than we may have initially recognized. For example, while adverse events in sepsis were initially thought to be due to exaggerated, pro-inflammatory cytokine production (i.e. “the cytokine storm”), increasing evidence supports an emerging hypothesis that the immunosuppression following the development of early sepsis contributes significantly to later complications of organ failure and mortality in sepsis [13]. As part of this shift in thinking, many investigators and clinicians now consider sepsis as having two overlapping phases. These phases may also occur concomitantly with both pro- and anti-inflammatory responses evident from the onset of sepsis [26]. An understanding of these phases helps guide research efforts as well as clinical care decisions.

The first phase, called the systemic inflammatory response syndrome (SIRS), is characterized by injurious, systemic inflammation and lasts several days following the onset of infection. SIRS develops when exaggerated immune cell activation responses damage host tissues and organs during efforts to clear infection. For example, pro-inflammatory cytokines synthesized by innate immune cells such as circulating monocytes and macrophages, as well as cells residing within tissues or organ compartments may augment host defense mechanisms against invading pathogens, but in doing so, also impair adaptive responses by immune and non-immune cells [27,28]. Clinically, SIRS is manifested as alterations in temperature (hypothermia or hyperthermia), tachycardia, tachypnea, and abnormal white blood cells counts (leukopenia or leukocytosis) [29].

The second phase, known as the compensatory anti-inflammatory response syndrome (CARS), may last anywhere from days to weeks. During the CARS phase, the immune system in some, but not all cellular compartments, is markedly suppressed, leading to secondary infection and organ failure [30]. As one example of this immunosenescence, immune cells isolated from septic non-survivors exhibit markers of immunosuppression and apoptosis. Moreover, the cells that remain demonstrate impairments in cytokine production, immune signaling, and associated innate and adaptive immune functions [13,31,32]. Recent evidence points to the immune suppression during CARS as a major cause of morbidity and mortality in patients with sepsis, although substantial knowledge gaps on this topic remain and in experimental animal models, the absence of lymphocytes, IL-10, and myeloid-derived suppressor cells may be protective [31,33-35].

These emerging discoveries have many important implications for the treatment of sepsis. Nevertheless, translating these findings to clinical care is challenging. These two phases often overlap, creating a highly complex and dynamic spectrum of pathophysiologic responses that may not be easily amenable to safe, effective therapeutic interventions [13,36]. Investigations are currently underway to parse out these complexities, and many biomarkers have been identified to describe these phases of treatment. For a more in-depth and well written review discussing these biomarkers and their implications and roles on future sepsis research the reader is referred elsewhere [37].

There is also increasing recognition that dysregulated immune cell functioning in sepsis is not due simply to alteration in one cytokine or one cell population. Rather, changes in a repertoire of pro- and anti-inflammatory cytokines, complement pathway mediators, coagulation factors, adipokines, and vascular permeability factors act in concert to cause much of the pathophysiology of sepsis [38]. During septic syndromes, one component of the immune system (e.g. a specific cytokine or immune cell) may be overly activated, causing injurious responses in the host. Yet, at other times during the course of sepsis, this same component may be deficient or have impaired functional responses, thus preventing appropriate host defense mechanisms. Taken together, these and other key findings have hindered our understanding of how to treat these heterogeneous and dynamic phases of sepsis.

### Immune cells mediate host reponses during sepsis

**Figure 1:**
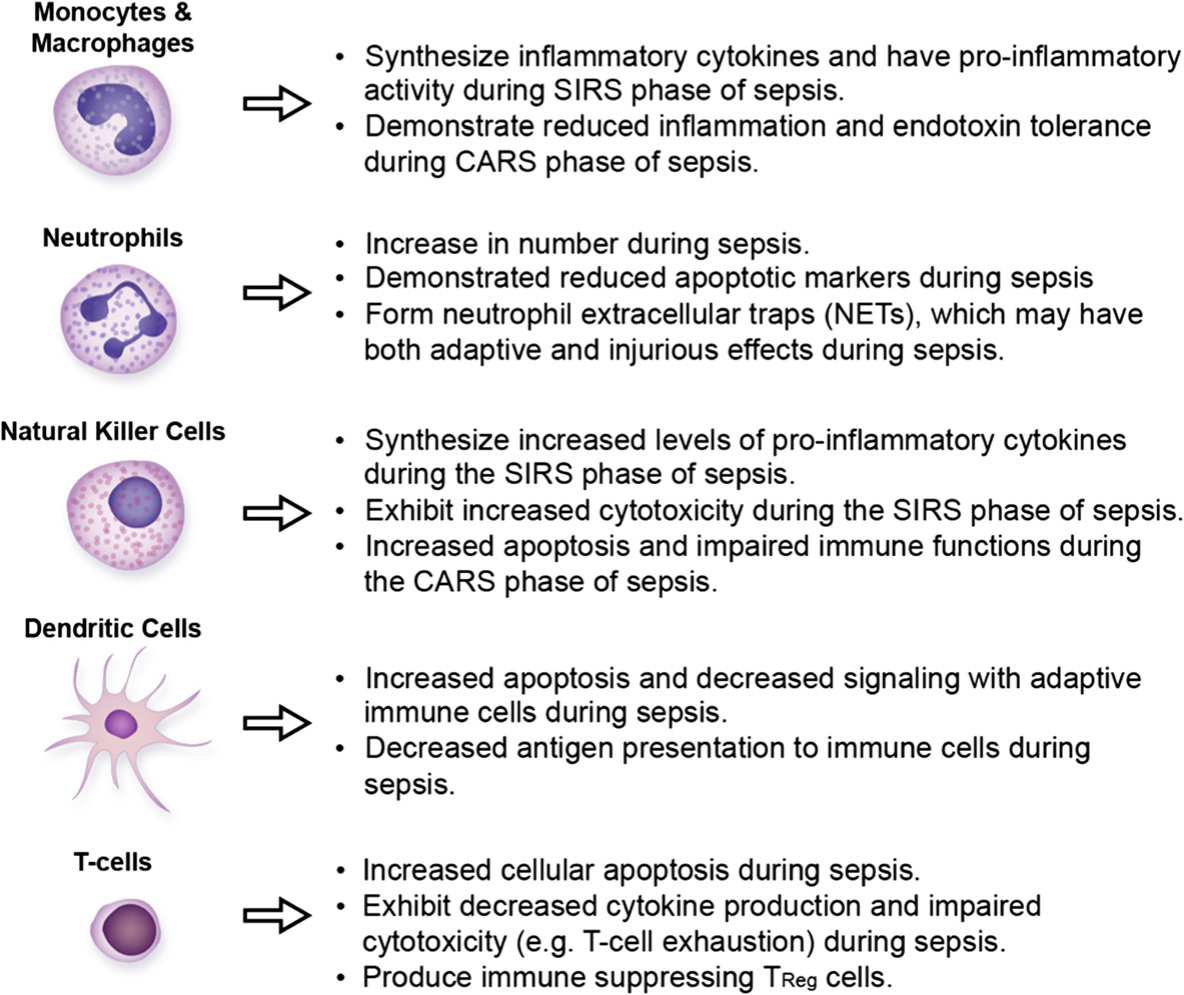
**Brief summary of some of the roles and functions of immune cells during septic syndromes.**

Although scientific advances continue, there remain many gaps in our understanding of immune cell functions and how they impact host responses during sepsis. Here, we briefly review some of these cells, their known functions during sepsis, and highlight several current challenges in understanding the role and contribution of these cells to the physiology and pathophysiology of sepsis (Figure 1). For further information on macrophages, monocytes, and natural killer cells, as well as the cellular subsets described briefly below, the reader is referred to several recent articles [13,39-42].

Polymorphonuclear neutrophils (PMNs) are a key arm of the innate immune response, and during sepsis PMN functioning is dysregulated [39,40]. While PMNs increase in number and demonstrate reduced markers of cellular apoptosis during sepsis [43], there is impaired migration of PMNs to areas of infection and misdirected accumulation within remote organ compartments [40,44]. These injurious, dysregulated responses correlate with sepsis-related morbidity and mortality, thus suggesting that alterations in PMN functioning during sepsis impact clinical outcomes [45].

Upon stimulation with lipopolysaccharide (LPS), direct microbial contact, or other agonists present within the septic milieu, PMNs also decondense and extrude their DNA into the extracellular space, forming neutrophil extracellular traps (NETs) comprised of nuclear chromatin, extracellular histones, and antimicrobial proteins [39,46,47]. Intriguingly, platelet toll-like receptor 4 (TLR4) [48] and platelet-derived human β-defensin 1 (hBD-1) [49] also induce NET formation, suggesting that platelets serve as immune sensors and activators during infectious insults.

The role and functions of NETs are still incompletely understood, but established and emerging evidence implicates NETs as key mediators of immune, inflammatory, and thrombotic pathways. Moreover, in some settings NET formation may augment host defense mechanisms, while in other situations NET formation may be injurious. For example, NETs mediate bacterial capture as well as interactions between bacteria and antimicrobial factors, enhancing bactericidal activity [39,46]. In premature neonates who are at increased risk of sepsis, NET formation is markedly impaired [50]. Nevertheless, NETs may have injurious effects, causing misdirected inflammation, thrombosis, and tissue damage [51-53]. Extracellular histones, a marker of NET formation, is cytotoxic on the endothelium, and in vivo, has been associated with organ failure and mortality in sepsis syndromes [54].

Dendritic cells (DCs) are a group of antigen-presenting cells (APCs) that interact with T and B cells, mediating key host defenses to pathogens and thus serving as a bridge between innate and adaptive immune responses. In sepsis, DC apoptosis is markedly increased. In this fashion, DCs may be a substantial contributor to the immunosenescence that characterizes the CARS phase of sepsis [55]. Nevertheless, a comprehensive understanding of DC functions in sepsis remains limited. Murine models have helped fill gaps in our understanding and demonstrated how augmenting DC function and number improve mortality following induction of endotoxemia, but these results have yet to be replicated in clinical settings [56]. Since dendritic cells have a major role in innate and adaptive immunity, DC apoptosis has potentially broad implications for developing new therapeutics in sepsis. Additionally, a better understanding of the mechanisms controlling dendritic cell death may help prevent sepsis-related morbidity and mortality [13,57].

In adaptive immunity, apoptosis of B and T cells also plays a critical role in host defense mechanisms during the SIRS and CARS phases. This has consequences on innate cell recruitment as well as adaptive cell function. Thus, understanding how to prevent or reverse B and T cell apoptosis may lead to new therapies for sepsis. Furthermore, if they do not undergo apoptosis, T cells may exhibit a phenomenon known as T-cell exhaustion. Only recently identified in septic syndromes, T-cell exhaustion occurs when cells are exposed to long-term and high antigen loads. The T cells subsequently have impaired cytokine production, are less cytotoxic, and are more apoptotic [13,31]. Currently, our understanding of the mechanisms inducing or regulating T-cell exhaustion is limited. Much work remains in order to understand how T-cell exhaustion can be prevented or reversed. Additionally, there is a subclass of CD4 + CD25+ T lymphocytes, known as T_Reg_ cells that are upregulated in sepsis [58,59]. T_Reg_ cells have several immune-suppressing effects, including some that are exhibited on monocytes [60]. However, what leads to T_Reg_ cell up regulation and control is still unclear. Moreover, other classes of T lymphocytes (e.g. CD4 + CD25-) are reduced in sepsis, highlighting the need for additional studies in this area.

### Animal models for sepsis

The use of animal models of sepsis has led to numerous new observations and discoveries, providing in vivo rationale for studies in humans. More recently there has been an increased appreciation for translating findings in sepsis animal models to human studies, although trials may be more limited than previously recognized. Despite decades of research and many preclinical trials utilizing well-defined and accepted animal models of sepsis, only a small number of agents and techniques have ultimately been demonstrated to improve the care of septic patients [61].

The reasons underlying this more limited correlation between animal and human settings of sepsis, which may not be surprising to some investigators, are not entirely understood. However, animal models often involve controlled, single insults that may not entirely recapitulate the natural history of sepsis in humans, where multiple infectious pathogens, wide differences in age, comorbidities, and therapeutic interventions are common. In addition, genomic responses to inflammatory insults may not correlate well between humans and mice, although these apparent differences are still not well understood [62,63] and recent studies have suggested that under some experimental conditions, gene expression patterns in mice are similar to those of human inflammatory settings [64]. Finally, a frequently used experimental animal model of polymicrobial sepsis, the cecal ligation and puncture (CLP) model, may not recapitulate clinical septic syndromes and emerging strategies to improve upon these models are being developed [65].

Despite these potential limitations, animal models currently remain an important tool in our arsenal for better understanding cellular responses in sepsis. Many observations seen in humans can be directly observed and correlated in mouse animal models [13]. As just one example, the widespread immune cell apoptosis observed in human sepsis is also observed in mouse models [66]. Thus, while in vivo models will continue to be utilized for studies investigating cell function, immune responses, and potential therapies in sepsis, we need to remain cognizant of the limitations of animal models when translating our findings to the human condition. Models that accurately mimic the physiologic, cellular, and molecular changes observed in human sepsis are difficult to achieve, yet remain an important goal in our journey to develop novel and effective therapies in sepsis.

## Conclusions

Sepsis remains a significant public health burden in the United States and worldwide. An understanding of the role of immune cells in the pathophysiology of sepsis remains limited but advances continue to be made, filling key knowledge gaps and identifying new potential therapeutic targets. Additional well-designed translational studies in sepsis are critical for success in this arena.

## Abbreviations

APC: Activated protein C
SIRS: Systemic inflammatory response syndrome
CARS: Compensatory anti-inflammatory response syndrome
PMNs: Polymorphonuclear neutrophils
LPS: Lipopolysaccharide
LPS: Neutrophil extracellular traps
TLR4: Platelet toll-like receptor 4
hBD-1: Platelet-derived human β-defensin 1
DCs: Dendritic cells
APCs: Antigen-presenting cells
